# Intramuscular Injection of Combined Calf Blood Compound (CFC) and Homeopathic Drug Tr14 Accelerates Muscle Regeneration In Vivo

**DOI:** 10.3390/ijms21062112

**Published:** 2020-03-19

**Authors:** Patrick Belikan, Lisa Nauth, Lars-Christopher Färber, Frédéric Abel, Eva Langendorf, Philipp Drees, Pol Maria Rommens, Ulrike Ritz, Stefan G. Mattyasovszky

**Affiliations:** Department of Orthopedics and Traumatology, University Medical Center of the Johannes Gutenberg University Mainz, 55122 Mainz, Germany; Patrick.Belikan@unimedizin-mainz.de (P.B.); lnauth@students.uni-mainz.de (L.N.); lars-christopher.faerber@unimedizin-mainz.de (L.-C.F.); Frederic.abel@unimedizin-mainz.de (F.A.); eva.langendorf@unimedizin-mainz.de (E.L.); Philipp.Drees@unimedizin-mainz.de (P.D.); Pol.rommens@unimedizin-mainz.de (P.M.R.); stefan.mattyasovszky@gmx.de (S.G.M.)

**Keywords:** muscle injury, calf blood compound, homeopathic drug, muscle regeneration, intramuscular injections

## Abstract

Skeletal muscle injuries in competitive sports cause lengthy absences of athletes from tournaments. This is of tremendous competitive and economic relevance for both the athletes and their respective clubs. Therapy for structural muscle lesions aims to promote regeneration and fast-track return-to-play. A common clinical treatment strategy for muscle injuries is the intramuscular injection of calf blood compound and the homeopathic drug, Tr14. Although the combination of these two agents was reported to reduce recovery time, the regulatory mechanism whereby this occurs remains unknown. In this in vivo study, we selected a rat model of mechanical muscle injury to investigate the effect of this combination therapy on muscle regeneration. Gene expression analysis and histological images revealed that this combined intramuscular injection for muscle lesions can enhance the expression of pro-myogenic genes and proteins and accelerate muscle regeneration. These findings are novel and depict the positive effects of calf blood compound and the homeopathic drug, Tr14, which are utilized in the field of Sports medicine.

## 1. Introduction

Skeletal muscle injuries are very common in sports, especially in professional sports. The incidence of muscle injury was reported to be as high as 28% in track and field/athletics [1] and 31% in soccer [2]. In a European soccer elite team, 15 muscle injuries per season can be expected to lead to the absence of players for more than 220 days of training and 37 matches [2]. As lower rates of injury during the sports season lead to better performances of professional soccer teams in their respective competitions [3,4], their medical staff is pressured to ensure their return to practice and competition at the earliest time attainable. Although skeletal muscle injuries have been considered a major concern in Sports medicine for several decades, treatment options remain very limited. To date, an international consensus on management in Sports medicine has not been derived. However, a well-accepted acute treatment strategy of injured skeletal muscles is the “RICE” principle, which comprises rest, ice (cold), compression, and elevation [5,6].

The primary and lasting aim of therapy is to enhance muscle healing and limit scar formation. Several injection therapies have been reported to accelerate recovery time and decrease the risk of recurrence [7,8]. Successful injection therapies of calf blood compound (CFC), a homeopathic drug (Tr14), local anesthetics, or platelet-rich plasma (PRP) has been frequently reported [7,8,9,10,11,12]. However, their underlying mechanism is unknown and their ability to shorten the time to return to sports has not been convincingly proven in large-scale randomized controlled trials [13]. CFC is a calf blood hemodialysate containing high concentrations of different amino acids, electrolytes, nucleosides, and trace elements; hence, defining a single pharmacologically active ingredient remains a challenge [13,14]. Previously published data have suggested the beneficial effects of CFC on numerous disorders, including acute and chronic wounds and malfunctions of blood circulation [15]. CFC has neuroprotective and anti-oxidative properties [16] and is meant to have ergogenic qualities and play an important role in muscle tissue metabolism [14]. CFC improves muscle cell proliferation and has been successfully applied in combination with Tr14 for the treatment of muscle injuries in the clinic [13].

Tr14 is a homeopathic solution that possesses anti-edematous, anti-exudative, and anti-inflammatory properties [17]. Tr14 consists of a combination of two minerals and twelve botanical substances. It inhibits the secretion of pro-inflammatory cytokines, such as IL-1β, TNF-α, and IL-8, by immunocytes [18]. The oral application of Tr14 has been demonstrated to have beneficial effects on epicondylitis and different musculoskeletal disorders [19,20]. Although numerous biochemical and cellular pathways seem to be modulated by its ingredients, the precise mechanism of action of Tr14 has never been explored in detail.

Although athletes are reported to be successfully treated in practice [19,21,22], only limited scientific evidence exists to support the general use of the above-mentioned agents in the treatment of skeletal muscle injuries in athletes. In a previous study conducted in our laboratory, we could prove in vitro that neither CFC nor Tr14 is myotoxic and that both could modulate the biological function of primary human skeletal muscle cells [23].

In the current in vivo study, we aimed to explore the regulatory impact of CFC and Tr14 on the healing process in a rat model with major structural skeletal muscle injury.

## 2. Results

### 2.1. Combined Intramuscular Application of Calf Blood Compound (CFC) and Tr14 Alters Gene Expression in Muscle Tears

As CFC and Tr14 were demonstrated to be non-myotoxic, we sought to evaluate their regulatory capacity on gene expression in an in vivo rat model. As a result, we opted to employ an established structural muscle injury model. Muscle injury in the quadriceps femoris muscle of Wistar rats was surgically created and treated with an intramuscular administration of sodium chloride, CFC, Tr14, or a combination of CFC and Tr14 into the lesions. At specific time points, rats were sacrificed, and the gene expression level of myosin heavy chain 1 (Myh1), neuronal cell adhesion molecule (NCAM), and paired box factor 7 (Pax7) of the injured muscle was determined.

**Figure 1:**
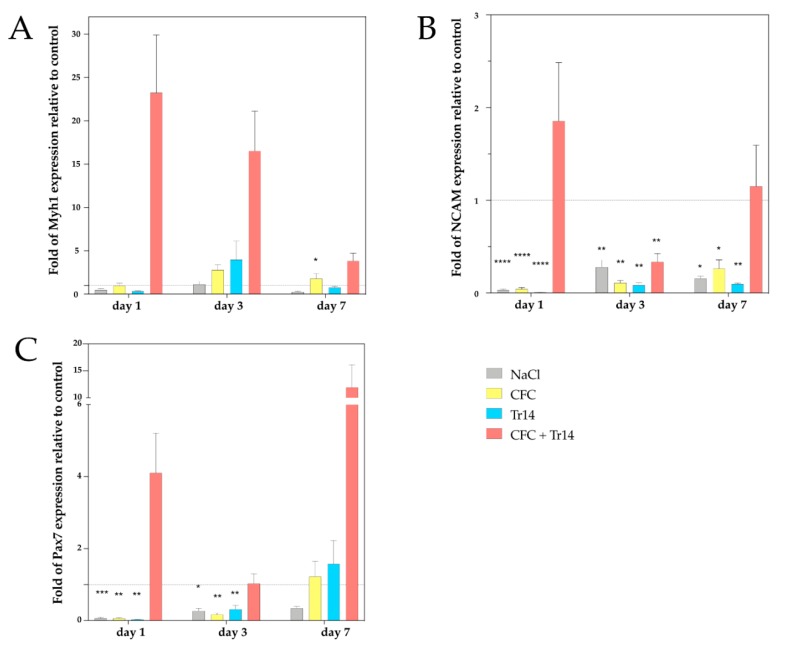
Gene expression analysis of myosin heavy chain 1 (Myh1) (**A**), neuronal cell adhesion molecule (NCAM) (**B**), and Pax7 (**C**) by qPCR at days 1, 3, and 7 following injury. The mRNA values of the treated groups were normalized to the housekeeping gene, 18S, and calculated relative to the untreated control group (marked as the interrupted line). Representative data of four different individuals in triplicate (total number of measurements, *n* = 12) are shown with SEM. P values < 0.05 indicated statistical significance. * *p* < 0.05, ** *p* < 0.01, *** *p* < 0.001, **** *p* < 0.0001.

On day 1 following muscle injury, animals administered the combination of CFC and Tr14 had a 23-fold higher Myh1 expression than the untreated control group (see Figure 1). Conversely, those treated with either sodium chloride, CFC, or Tr14 did not display Myh1 upregulation on day 1 after surgery. By comparing the applied treatments on day 1, we could identify differences in Myh1 expression in the group treated with the combination of CFC and Tr14 compared to the groups administered Tr14 and sodium chloride, respectively.

Although Myh1 was upregulated three days after injury in animals treated with CFC and Tr14 as single or combined therapy, we found no statistically significant differences between these groups. Seven days after injury, the expression of Myh1 decreased in rats administered combination therapy. However, a higher Myh1 expression was found in rats treated with both CFC and Tr14 compared to those administered either CFC, Tr14, or sodium chloride.

In the group treated with a combination of CFC and Tr14, the gene expression level of NCAM was upregulated on day 1 and slightly upregulated on day 7 after surgery. In contrast, NCAM expression level on day 4 post-surgery was downregulated in animals administered combination therapy compared to the non-treated control groups. In the remaining groups, NCAM was significantly downregulated at all time points (Figure 1). By comparing the individual treatment groups, CFC administered in combination with Tr14 resulted in a higher NCAM expression than the monotherapies on days 1 and 7 (no statistical significance).

The expression level of the satellite cell (SC) marker, Pax7, was downregulated in the monotherapy groups on days 1 and 3, but upregulated in the combined therapy group on days 1 and 7 and in the CFC and Tr14 monotherapy groups on day 7. Compared to animals treated with sodium chloride, or the CFC or Tr14 monotherapy, those administered CFC in combination with Tr14 had enhanced Pax7 expression on days 1 and 7.

In summary, muscle lesions treated with the combined therapy of CFC and Tr14 demonstrated alterations in their gene expression patterns compared to lesions treated with the respective monotherapies.

### 2.2. Accelerated Muscle Regeneration after Combined Therapy with CFC and Tr14

To characterize the impact of intramuscular injections of CFC and TR14 on the muscle healing process, we performed a histological analysis of muscle lesions in the quadriceps femoris muscle treated with intramuscular injections of CFC and Tr14 in the site of lesion at different time points.

Over time, evident histological differences could be identified among the different groups. In subsequent experiments, we focused on the differences in the groups at the respective time points.

**Figure 2:**
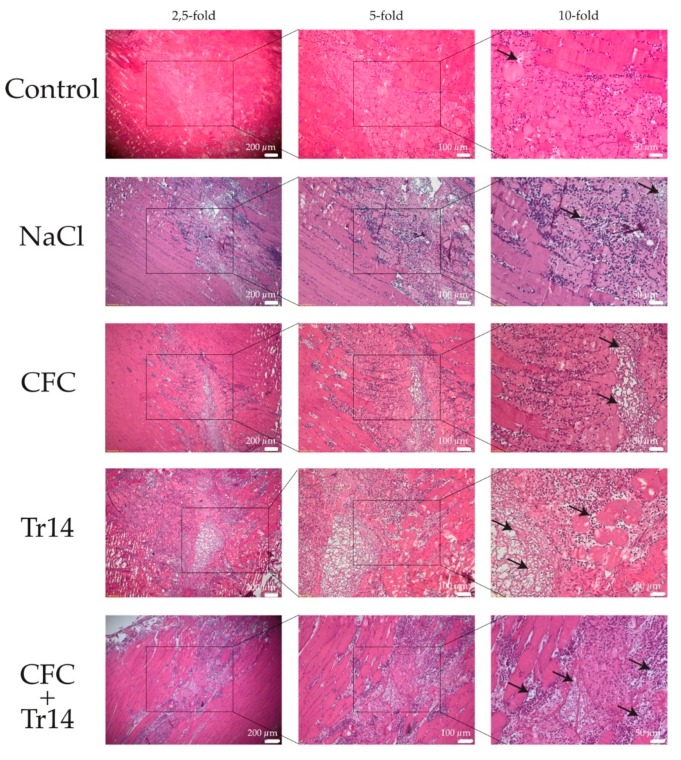
Histological sections at day 1 after surgery. By HE-staining, an increased number of mononucleated cells and more edema (black arrows) were observed in the treated groups compared to the control group. The boxes in the first columns show the area magnified in column 2 and the boxes in column 2 show the area magnified in column 3.

One day after surgery, a distinct amount of tissue edema and necrotic myofibers, connective tissue, and blood vessels could be detected in the site of the lesion (Figure 2). Additionally, there was no evident difference between the histomorphology of the lesions among the examined groups. However, degeneration in the control group was not as apparent as that in the treated groups, presenting less tissue edema. Histological sections of animals treated with Tr14 or CFC+Tr14 presented higher amounts of mononucleated cells in the site of the lesion compared to the control group and the sodium chloride group (Table 1). There was no visible difference in the size of the necrotic areas on day 1 (Figure 2).

**Figure 3:**
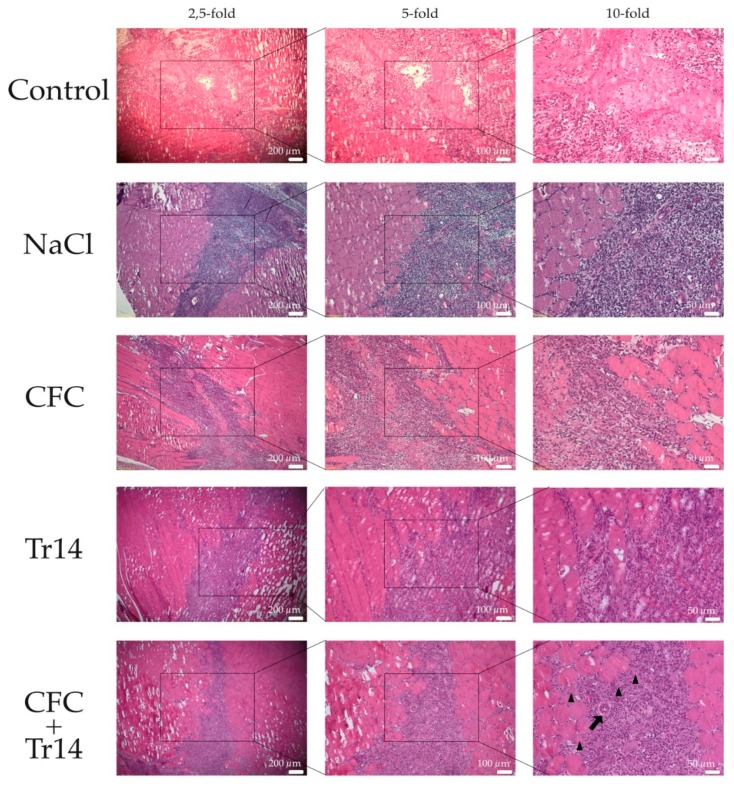
Histological sections at day 3 after surgery. By HE-staining, inflammatory infiltrates were observed in all groups. Isolated regenerated myofibers (black triangles) and newly formed arteriole (black arrow) were observed in muscles treated with the combined therapy. The boxes in the first columns show the area magnified in column 2 and the boxes in column 2 show the area magnified in column 3.

**Figure 4:**
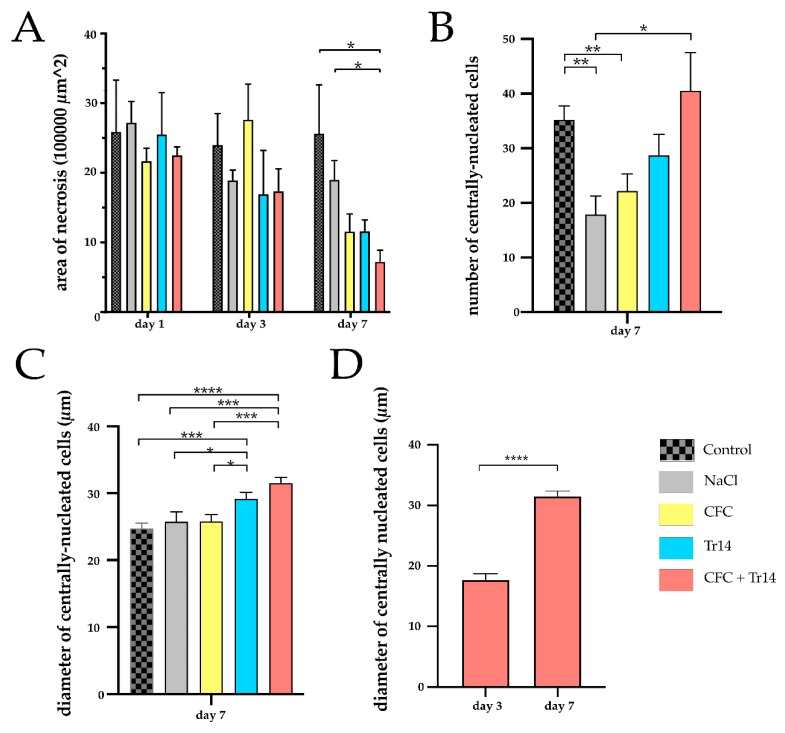
Quantitative histological analyses for the site of laceration. (**A**) Necrotic area of the injured muscle at days 1, 3, and 7 following surgery in the experimental groups. (**B**) Numbers of centrally nucleated, regenerative cells per sample at day 7 following muscle injury. (**C**) Diameter of regenerative cells in the different groups at day 7. (**D**) Comparison of the diameter of centrally nucleated cells on day 3 and day 7 in rats treated with CFC + Tr14. Data are presented as mean + SEM. * *p* < 0.05, ** *p* < 0.01, *** *p* < 0.001, **** *p* < 0.0001.

In contrast to the first day, all groups were characterized by an increase in leukocyte infiltration at three days post-surgery (Figure 3 and Table 1). Although edema decreased in the treated groups, it became more evident in the untreated control. There was no difference in the necrotic areas within the groups (Figure 4A). In animals treated with the combination of CFC and Tr14, regenerating myofibers at the periphery of the necrotic tissue area were observed (*n* = 8 per observation field, data not shown), which appeared like an incipient regenerative zone [24]. No signs of the so-called regenerative zone were observed in the other groups. Isolated regenerating myofibers could also be found in the Tr14 group (*n* = 2 per observation field, data not shown).

**Figure 5:**
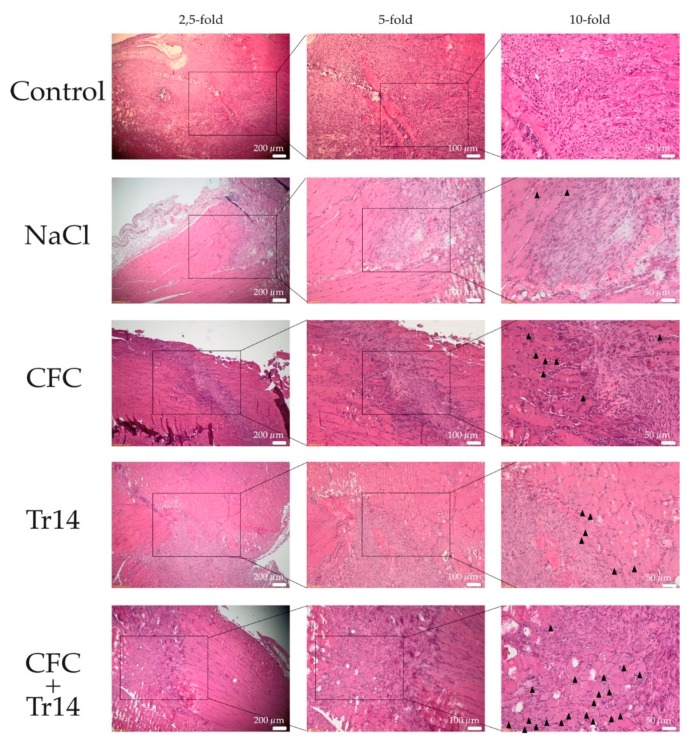
Histological sections at day 7 following surgery. By HE-staining, the accelerated regeneration in the group treated with calf blood compound (CFC) only or CFC combined with Tr14 was revealed relative to the control group, sodium chloride group, and the Tr14 group. Regenerated myofibers with centralized nuclei are highlighted (black triangles). The boxes in the first columns show the area magnified in column 2 and the boxes in column 2 show the area magnified in column 3.

Seven days after surgery, the regenerative myofibers could be observed in all groups (Figure 5). The highest number of centrally nucleated cells was detected in animals treated with the combined therapy and the control group (Figure 4B). Large areas of myocyte necrosis with lower numbers of infiltrating leukocytes were observed in all groups compared to the sections retrieved three days after surgery. Compared to the groups treated with Tr14 alone or CFC and Tr14 combined, leukocyte infiltration in the control group, sodium chloride, and CFC group was pronounced (Figure 5 and Table 1). Irrespective of groups, there was no visible tissue edema seven days after surgery. In animals administered the combination therapy, their regeneration zone expanded and only a small necrotic zone was apparent. This effect was significantly decreased relative to day 3 (*p* = 0.029, data not shown). Furthermore, the size of the necrotic area in rats treated with the combination of CFC and Tr14 was significantly lower than that in animals of the control group and the sodium chloride group (Figure 4A). Animals administered either of the single injection therapy tended to exhibit a decrease in the size of their necrotic area compared to the untreated control group and the sodium chloride group.

The diameter of the newly formed myofibers was demonstrated to increase in the order: from the control, sodium chloride, CFC-treated, Tr14-treated, to the combined therapy group (Figure 4C). Compared to day 3, on day 7, larger diameters of centrally nucleated myofibers could be observed in the group treated with the combined therapy (CFC + Tr14) (Figure 4D).

To summarize, muscle fiber regeneration in animals treated with CFC and the homeopathic drug Tr14 appeared to be accelerated, displaying the highest number of centrally nucleated cells and small necrotic areas. Arteriole formation was only observed in animals administered the combination therapy, indicating its capability to induce new vessel formation (Figure 3).

**Figure 6:**
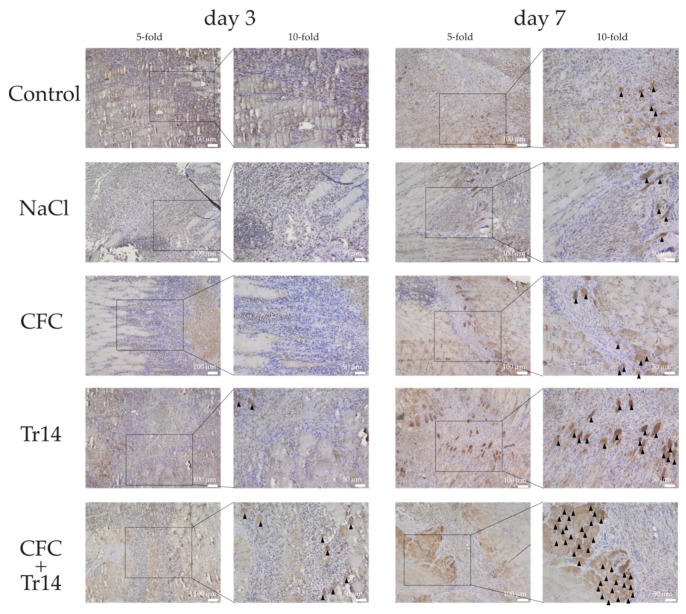
Immunohistological sections stained for myogenin positive cells at day 3 and 7 following surgery. Regenerated myofibers with high myogenin expression are highlighted (black triangles). The boxes in the first columns show the area magnified in column 2.

As myogenin represents one important factor in muscle differentiation [25,26], immunohistological stainings for myogenin were performed at days 3 and 7 following surgery (Figure 6). Immunohistological stainings for myogenin support the results of the HE stainings. The highest number of myogenin positive cells was detected in animals treated with the combined therapy, followed by the Tr14 group and CFC group after 7 days. Three days after surgery, a thin regenerative zone of newly formed myofibers could be observed in the animals treated with the combined therapy.

## 3. Discussion

For more than three decades, health professionals and scientists have attempted to accelerate muscle healing. Different injection methods have been reported to serve as non-surgical treatment options that can reduce the recovery time following strain injuries [6,8,12]. However, further studies negated the beneficial properties of some of these therapies [7,27,28].

Intramuscular application of CFC in combination with Tr14 is a very common treatment strategy that has been successfully applied by sports medicine doctors for more than 20 years [13,29]. The treatment of muscle tears with intramuscular CFC application was first described by Pfister et al. [12]. Through a double-blind randomized control trial, Pfister and colleagues demonstrated that CFC led to a reduction in the rehabilitation time compared to the placebo. The treatment regimen described years later, which include the dual application of CFC and Tr14 on days 0, 2, and 4 after injury, is expected to decrease the return-to-play of athletes [19], enabling. This indicated that CFC and Tr14 may accelerate healing of connective tissue and support the formation of new skeletal muscle fibers in the recovery of structural muscle injuries. Intramuscular CFC application was first described by Pfister et al. They could illustrate in a double-blind randomized control trial that CFC lead to a reduction of rehabilitation time compared to a placebo group. The more common treatment regimen described a few years later with the application of CFC and Tr14 in combination on day zero, two, and four after injury was described to result in a faster return-to-practice and return-to-play [19]. Tr14 was also demonstrated to have bio-regulatory effects in the treatment of musculoskeletal injuries in exercise induced inflammation [29,30,31,32], and comparable effectiveness to NSAIDs for the reduction in symptoms of inflammation [29].

The above-mentioned data suggest that CFC and Tr14 may accelerate the healing process of injured connective tissue. To our knowledge, this is the first study to examine the effect of the intramuscular application of the combination of CFC and Tr14 on skeletal muscle healing in vivo.

### 3.1. Intramuscular Application of the Combination of CFC and Tr14 Alters Gene Expression in Muscle Tears

Myosin heavy chain-1 (Myh1) is a pro-myogenic skeletal muscle-specific gene that is required for muscle assembly and function [33,34]. Regenerating myofibers in a rat model were demonstrated to express high amounts of Myh1 in the early stages following muscle injury; however, on day 4, its expression level decreases [35], which aligns with our findings. In our study, we found a remarkable upregulation of Myh1 on day 1 after injury (one treatment of CFC in combination with Tr14) and a downregulation throughout the remaining course of the study. The increased expression of the myogenic marker, Myh1, upon treatment with a combination of CFC and Tr14 in the early phase following muscle injury indicates the pro-myogenic effect of the two agents.

Another marker of muscle regeneration is NCAM, which is expressed in the myoblasts, muscle fibers, and myotubes [36,37,38,39]. In our experiment, the expression level of NCAM was upregulated on day 1 but to a lower level on day 7 in animals treated with the combined therapy (CFC + Tr14). Upon treatment with sodium chloride, single CFC, or single Tr14, the expression level of NCAM was downregulated at all examined time points. Such a finding is consistent with the results of increased NCAM expression in regenerating muscle cells after extracorporeal shock wave therapy (ESWT) on a muscle lesion [40].

An important hallmark of skeletal muscle is regeneration following damage. Regeneration is significantly influenced by the interaction between SCs and their microenvironment [41,42]. The process of regeneration is highly orchestrated and includes the activation and migration of SCs to the site of injury and their proliferation and differentiation into muscle fibers [43]. SCs are located between the sarcolemma of the muscle fiber and the basal lamina and are quiescent (G0 phase) during homeostasis. Upon injury, SCs are activated resulting in their re-entrance into the cell-cycle and their contribution to the formation of new muscle fibers [44,45]. Pax7 is expressed by all SCs in the skeletal muscle and is crucial for function [46,47,48,49,50]. In animals treated with a combination of CFC and Tr14 following muscle lesion, we observed an increase in the upregulation of Pax7 on days 1 and 7 compared to levels found in the control group. On day 3, no difference could be detected between the dual-treatment and the control group. The increased level of Pax7 expression on day 7 aligns with the findings of Fisher, with SCs being a prominent feature at this point. The decreased expression of Pax7 in the group administered the combination of CFC and Tr14 on day 3 seems contradictory to the accumulation of SCs in the area of muscle lesions three days after trauma [51]. The decreased expression observed at this time point can be explained by the accelerated muscle regeneration in rats with a myogenic response in the early phase of the regeneration process [52], the accompanied progress of differentiation of SCs, and concomitant downregulation of Pax7. Furthermore, the increased expression of Pax7, which reappeared on day 7, could result from the recurrence of quiescent and self-renewed SCs. Single treatment with either CFC, Tr14, or sodium chloride leads to significantly decreased Pax7 expression compared to combination treatment.

Based on these findings, we could illustrate that intramuscular injection of the combination of CFC and Tr14 modulates in vivo gene expression to a more regenerative typical phenotype.

### 3.2. Accelerated Muscle Regeneration after Combined Therapy with CFC and Tr14

Different muscle injuries have been induced in animal models in the recent years, such as freeze injury, myotoxin injury, and chemical injury using an intramuscular injection of barium chloride [24]. To evaluate the healing process in structural skeletal muscle injury, we sought to use a mechanical injury model with laceration of the quadriceps femoris muscle in rats, with modification to reflect the protocol of Nakaska et al. [53]. Surgical lesion models have been described to best represent injuries arising during the performance of sporting activities [54,55].

Muscle regeneration after injury follows three interrelated and time-dependent phases, namely the degeneration phase, the remodeling phase, and the maturation phase [44,46]. The phases are similar after different types of muscle damage and in different organisms; however, the amplitude and the kinetics differ between each organism and depend on the extent of damage [56].

The degenerative phase is characterized by necrosis of the muscle fibers and the connective tissue and is accompanied by infiltration of leukocytes, which is a necessary inflammatory process for the removal of necrotic cellular debris [56]. The greatest abundance of leukocytes can be observed two to four days after muscle damage [57]. This result aligns with the present findings as leukocyte accumulation was found to increase in the site of the lesion at day 3 following muscle injury in all groups.

The remodeling phase is induced by inflammation of the degenerative phase and is characterized by the activation and proliferation of myogenic cells and the formation of new myofibers. The myogenic cells, mainly SCs, migrate to the site of the lesion, differentiate and repair damaged myofibers, or fuse to form new myofibers [58]. The remodeling phase can last up to two weeks after muscle damage and is histopathologically characterized by newly formed basophilic myofibers [56]. Remodeling of the extracellular matrix and angiogenesis occur within this regeneration phase. In our study, we could observe the remodeling phase in all groups within the first seven days following muscle injury. However, there were clear differences between the groups. Only the group administered the combined therapy with CFC and Tr14 formed myofibers and demonstrated angiogenesis, with new arterioles at day 3 after surgery. At seven days following injury, there was no consistent pattern between the groups, and regeneration was found to be most advanced in the combined therapy group. However, in the single Tr14 group, a remarkably advanced regeneration, but with more persisting cell debris, could be observed. The CFC and the sodium chloride monotherapy groups had less newly formed myofibers; however, compared to the untreated group, their numbers were increased.

By day 15, the maturation phase follows the remodeling phase [56] and at this point, fusion of cells is completed, the size of the newly formed myofibers increases, and the regenerated fibers are reinnervated. As our findings did not show an evident difference in the size of the newly formed myofibers between the groups, a valid statement regarding the maturation phase could not be presented based on our animal model; this is because injuries were only observed seven days after muscle injury.

To summarize, in the present study, we could demonstrate that compared to the single therapies with CFC, Tr14, or sodium chloride, the combination of CFC and Tr14 caused an earlier regeneration onset. This can be explained by the suggestion that CFC improves endothelial function owing to its high amount of sphingolipids [59] and consecutive angiogenesis as demonstrated in our animal model. The interaction between vessels and myogenic cells is crucial for muscle function and regeneration, not only because of the supply of oxygen and nutrients, but also the direct interactions between endothelial cells and myogenic cells [60]. CFC was demonstrated to elicit effects on the cell’s energy metabolism. In animal models, CFC stimulates glucose uptake in cells. Moreover, it induces a protective effect during hypoxia by enhancing oxygen uptake [17]. By decreasing reactive oxygen species (ROS), which are produced as a result of ischemia and inflammation, CFC protects cells of multiple organs and tissues [61]. These mechanisms could explain the protective properties of CFC injection therapy as observed clinically and in our animal studies following muscle tears. The ability of Tr14 to reduce inflammation [29,30,31,32] could be the potentiating effect that accelerates regeneration in the group administered the combined CFC and Tr14 therapy.

### 3.3. Limitations of the Study

Although our results are supported by different experiments and results, the present study had some limitations.

First, we focused on the gene expression of some myogenic transformation factors. Using more transformation factors, such as MyoD, Myf 5, and MyoG, could have led to more information on specific cells. Furthermore, fibrotic, fatty, or inflammatory genes such as transcription factors from the COX pathway and/or angiogenic factors could have given further information. Nonetheless, they will be included in our follow-up study.

The therapy schedule can be considered as another limitation. We opted to transfer the treatment regimen used in humans to our in vivo rat model. As there are differences between the regeneration processes of the two species, other therapy schedules with different timepoints and frequencies of injection, as well as different drug concentrations might influence the regenerative effects.

With the seven-day observation period, the maturation phase did not begin, and regeneration was incomplete. Therefore, a definitive conclusion could not be drawn regarding the influence of the combined CFC and Tr14 therapy on the maturation phase and the finalized muscle regeneration

### 3.4. Conclusions

To our knowledge, this is the first in vivo study to evaluate the effect of the intramuscular application of a combination of CFC and the homeopathic drug, Tr14, in the treatment of structural skeletal muscle injuries.

The combined application of CFC and Tr14 resulted in an increased number of newly formed myofibers at the site of the lesion and a higher expression of myogenic genes, thereby supporting the combined use of CFC and Tr14 as a therapeutic injection in the clinic for muscle injuries in sports. However, the underlying mechanisms remain unclear. Therefore, we recommend that further in vivo and clinical studies be conducted to confirm the results presented herein.

## 4. Materials and Methods

### 4.1. In Vivo Rat Model and Experimental Procedure

All animal experiments were approved by the local authorities (approval number G12-1-088, 03-13-2014) and conducted according to the German Animal Protection Law.

**Figure 7:**
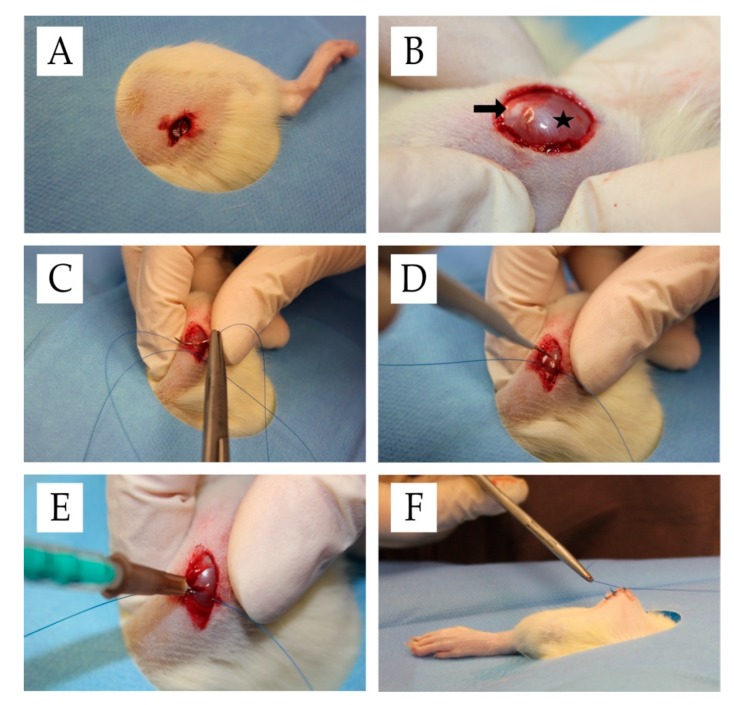
Surgical procedure. **(A)** Situs after skin incision. **(B)** Exposed rectus femoris muscle (star) and patella (arrow) after skin incision. **(C)** Filament-marking of the muscle lesion. **(D)** Lesion induction in the rectus femoris muscle. **(E)** Intraoperative intramuscular injection treatment. **(F)** Situs after cutaneous suture.

Wistar rats were obtained from Janvier Labs (France). Twelve-week-old male animals were kept in-house in single cages. Rats were allowed to acclimate to the 12-h light-dark cycle for at least seven days. A total of 150 animals underwent surgery. Thereafter, they were divided into five experimental groups (30 animals per group) and sacrificed at three time points (10 animals per group and time point; *n* = 10). The surgical procedure was performed as described below (Figure 7). Briefly, rats were initially anesthetized using isoflurane with a face mask and a subcutaneous injection containing midazolam (2 mg/kg), medetomidin (150 µg/kg), and fentanyl (5 μg/kg). After depilation, a skin incision of 1 cm between the lateral femoral condyle and the greater trochanter of the right thigh was performed. Thereafter, a 3-mm lesion (depth and width) was created with a knife (blade size 11) in the rectus femoris muscle transverse to fiber orientation. To identify the exact position for further percutaneous injections and post-mortem examinations, the inflicted lesion was marked at both ends with a 4-0 Safil-suture. The skin was closed using a 4-0-filament. On average, the duration of surgery was approximately 15 min. During surgery, body temperature was maintained at 35–37 °C. For histology, the marked muscle lesion was carefully excised. Muscle samples were either stored in RNAlater^®^ solution (Invitrogen Life Technologies Thermo Fisher Scientific, Carlsbad, CA, USA) for gene expression analyses or fixed in Roti^®^ Histofix-4.5% (Carl-Roth^®^ GmbH, Karlsruhe, Germany) for histological analyses. Rats were sacrificed at different time points by exposure to CO_2_.

### 4.2. Therapy Schedule

To determine the effects of intramuscular injections of CFC (Actovegin^®^ 200 mg/5 mL, Takeda, Linz, Austria) and the Homeopathic Drug, Tr14 (Traumeel^®^ S, Heel, Baden-Baden, Germany), rats intraoperatively received intramuscular injections of either sodium chloride, Actovegin^®^, Traumeel^®^ or both Actovegin^®^ and Traumeel^®^.

**Figure 8:**
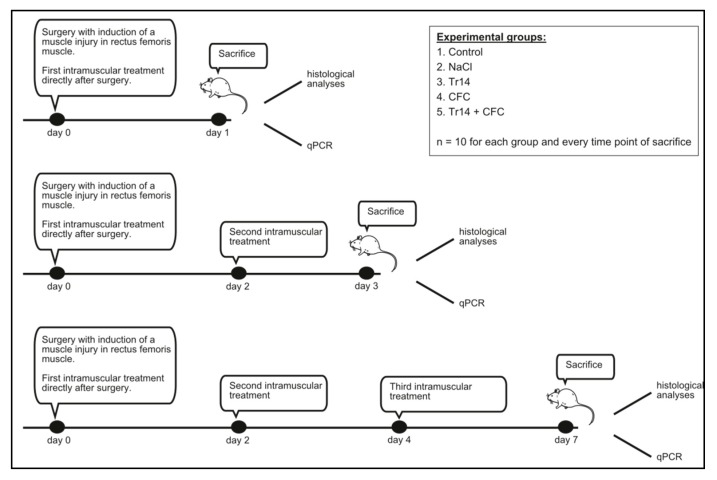
Intramuscular injections were performed according to the groups, with intramuscular injections of NaCl, CFC, Tr14, CFC + Tr14, or no injection in the control group (*n* = 10 for each group and time point). Injection volume was 100 µl. Injection concentrations were: 1) CFC (Actovegin^®^): 0.965 µg/g bodyweight (BW), Tr14; 2) and Tr14 (Traumeel^®^): 7.64 pl/g BW. Rats receiving both agents were treated in a 2:1 ratio (CFC:Tr14).

Injection volume was determined to be 0.1 mL. Sodium chloride injections were performed with physiological saline solution (0.9%) (Braun, Melsungen, Deutschland). The concentration for the CFC- and Tr14- injection was selected according to the injection protocol for human muscle [13]. The protocol recommended a 2-mL solution containing CFC and Tr14 in a 2:1 ratio for an 80-kg athlete. Accordingly, a concentration of 7.64 pL/g bodyweight (BW) Tr14 and 0.965 µg/g BW CFC was calculated to serve as the injection concentration in our animal model. The control group received no treatment after the induction of the muscle lesion; thus, physiological cure could be observed in this group of animals. Rats were treated and sacrificed according to Figure 8. The first group of animals was sacrificed on day 1 after surgery and intramuscular injection. The remaining rats received a second intramuscular injection according to their previous therapy. On day 3 following surgery, a second group of rats was killed. The last group of animals received a third intramuscular injection on day 4 and was sacrificed on day 7 following surgery. Re-injections were performed using isoflurane anesthesia with a face mask as previously described. Each experimental group consisted of 10 animals at each time point (*n* = 10).

The rat muscle tissue of each group was used for RNA isolation, and histological and histochemical analyses.

### 4.3. RNA Isolation, cDNA Synthesis, and qPCR

For RNA isolation, the muscle tissue between the two marking suture knots was extracted. After dousing with liquid nitrogen, the tissue was finely crushed. Using the RNAeasy Mini Kit (Qiagen, Venlo, Netherlands), 20 mg of each tissue sample was lysed and homogenized. After subsequent loading and binding with 70% ethanol and DNA digestion, RNA was isolated with the included RNA binding columns, according to manufacturer’s instructions. We reversed-transcribed 2 µg of RNA into cDNA using Super script^®^ III reverse transcriptase (Invitrogen™, Life Technologies, Thermo Fisher Scientific, Carlsbad, CA USA), Random Primers (Promega, Madison, Wis USA), and dNTPs (Bioron GmbH, Ludwigshafen, Germany). By conducting qPCR with the SYBR^®^ Green Master Mix (Applied Biosystems, Foster City, CA, USA) and QuantiTect primers (Qiagen, Hilden, Germany) (Table 2), the rate of gene expression were analyzed according to manufacturer’s instruction. Amplification conditions were 40 cycle numbers consisting of denaturation for 15 s at 95 °C, 30 s of annealing at 55 °C, and 35 s of extension at 72 °C. Measurement values were indicated as a fold expression of the housekeeping gene, 18S. All qPCR measurements were performed with RNA from four different individuals in triplicate (total number of measurements, *n* = 12). The results were calculated using the 2^−ΔΔCt^ method [62]. Measurement values were normalized using the housekeeping gene and indicated as fold expression compared to the untreated control.

Cell viability and gene expression of Pax7, NCAM, myogenic factor 5 (Myf5), and MyoD, which are muscle cell markers, were compared to those of non-treated muscle cells, which served as controls. Quiescent satellite cells express the paired-box transcription factor, Pax7, and when activated, they co-express Pax7 with a member of the myogenic regulatory factor (MRF) family, which consist of MyoD, Myf5, Myogenin, and MRF-4. Myf5 and MyoD were considered the early MRFs, while Myogenin and MRF-4 were considered to be the late MRFs. Myf5 is a direct target gene of Pax7, and the transcription of Myf5 directly varies with Pax7 levels.

NCAM is another marker of muscle regeneration that is expressed in myoblasts, muscle fibers, and myotubes. Myh1 is a pro-myogenic skeletal muscle-specific gene, which is required for muscle assembly and function. In a rat model, regenerating myofibers was demonstrated to express high amounts of Myh1 in the early stages following muscle injury; however, on day 4, its expression level decreases. In our gene expression experiments, we focused on early differentiation of cells. Based on the above information, our previous findings in muscle cell regeneration, and published data, we opted to investigate these three markers.

### 4.4. Histological Analyses

The muscle tissue was extracted and stored in 4% paraformaldehyde. After embedding in paraffin blocks (Roti^®^-Plast Paraffin, Carl-Roth^®^ GmbH, Karlsruhe, Germany), the fixed tissues were cut into 5-µm slices (Mikrotom 2030, Reichert-Jung, Heidelberg, Germany). Sections were then stained with Hematoxylin/Eosin.

### 4.5. Immunohistochemistry

The sections were first treated with citrate buffer (0.1 M citric acid, 0.1 M sodium citrate), then blocked for 20 min with 3% H_2_O_2_ followed by a 30 min incubation step with horse serum (Biochrom GmbH, Berlin, Germany). Myogenin mouse-monoclonal antibody (MyBioSource/Biozol MBS438443) was incubated overnight at 4 °C. The sections were first labeled with a biotinylated linker, followed by streptavidin-conjugated horseradish peroxidase, and finally counterstained with hematoxylin.

### 4.6. Morphometric Analyses

Two-dimensional analysis was performed using ImageJ (NIH, Rockville Pike, Bethesda, MA, USA). Necrotic area, number of centronucleated, regenerating myofibers within the lesion sites, and their diameter were evaluated. Three random fields within the lesion area were selected in each sample. The number of regenerative myofibers was expressed as the number of regenerative myofibers per sample. Evaluation was performed by two independent examiners (P.B. and L-C.F.).

### 4.7. Statistical Analysis

Statistical analysis was performed with GraphPad Prism Version 7 (GraphPad Software, San Diego, CA, USA) and IBM^®^ SPSS^®^ Statistics Version 23 (IBM Inc., Armonk, NY, USA). The results are presented as mean ± SEM. Normal distribution was evaluated using normal probability plots and the Shapiro-Wilk-Test. If data were considered to be non-normally distributed, univariate ANOVA (Kruskal-Wallis-Test) was employed followed by the Mann-Whitney-U-Test. If data were considered to be normally distributed, the unpaired *t*-test was performed. A *p* value < 0.05 was considered to indicate statistical significance.

## Figures and Tables

**Table 1:** Semi-quantitative analysis of leucocyte infiltration observed by histology.

Experimental Group	Day 1	Day 3	Day 7
Control	++	+++	++
Sodium chloride	++	++++	++
CFC	+++	+++	++
TR14	+++	++++	+
CFC + Tr14	+++	+++	+

Results are expressed as a percentage of the tissue infiltrated by inflammatory cells: (+) 10% to 30%; (++) 30% to 50%; (+++) 50% to 80%, (++++) more than 80%.

**Table 2:** The QuantiTect primers used for qPCR.

Product Name	Catalog No.
Rn_Myh1_1_SG QuantiTect Primer Assay	QT00384307
Rn_Pax7_1_SG QuantiTect Primer Assay	QT01301552
Rn_Ncam1_1_SG QuantiTect Primer Assay	QT00181944
Rn_Rnr1_1_SG QuantiTect Primer Assay	QT00199374

## Abbreviations

BW	Bodyweight
CFC	Calf Blood Compound
DNA	deoxyribonucleic acid
IM	Intramuscular
IL	Interleukin
kg	Kilogram
Mg	Milligram
MRF	myogenic regulatory factor
Myh1	Myosin heavy chain-1
NaCl	Sodium chloride
NCAM	Neuronal cell adhesion molecule
Pax	Paired box factor
PCR	Polymerase chain reaction
pl	Picolitre
PRP	Platelet-rich plasma
RNA	ribonucleic acid
s	second
SC	Satellite cell
TNF	Tumor necrosis factor
